# Inclusivity in Oral Care: A Comprehensive Dental Prevention Approach to Improve Cooperation and Oral Hygiene in Children with Special Needs

**DOI:** 10.3390/jcm15124580

**Published:** 2026-06-12

**Authors:** Dalma Tábi, Orsolya Németh, Bálint Zsombor Sárai, Péter Hársfalvi, Kornélia Farkas, Tímea Vissi, Péter Hegyi, Gábor Varga, Dániel Végh, Alexander Schulze Wenning, Ibolya Túri

**Affiliations:** 1 Department of Dental Public Health, Semmelweis University, 1088 Budapest, Hungary; tabidalma@gmail.com (D.T.);; 2 Centre for Translational Medicine, Semmelweis University, 1085 Budapest, Hungary; 3 Institute of Bioanalysis, Medical School, University of Pécs, 7624 Pécs, Hungary; 4 András Pető Faculty, Semmelweis University, 1125 Budapest, Hungary; 5 Institute for Translational Medicine, Medical School, University of Pécs, 7624 Pécs, Hungary; 6 Institute of Pancreatic Diseases, Semmelweis University, 1083 Budapest, Hungary; 7 Department of Oral Biology, Semmelweis University, 1089 Budapest, Hungary; 8 Department of Prosthodontic, Faculty of Dentistry, Semmelweis University, 1085 Budapest, Hungary

**Keywords:** special needs, oral health, children, cooperation, preventive education

## Abstract

**Objectives:** Special care dentistry provides essential, customized oral health care for individuals with significant disabilities, addressing higher rates of dental issues and enhancing overall well-being. The aim of this pilot study was to evaluate the effect of an eight-week dental prevention program on oral health outcomes and cooperation during dental treatment in children with special needs. **Methods:** The study targeted 97 children from Pető András Conductive Practical Primary School. A total of 16 children participated in the program, which consisted of eight weeks of education sessions focusing on oral hygiene practices and dental cooperation strategies. Dental assessments were conducted at baseline, weeks 8, 16, 52 and 104 using WHO protocols. **Results:** Significant improvements were observed following the 8-week preventive education program. OHI-S, DI-S, and CI-S values showed significant reductions at both week 8 and week 16 compared to baseline (*p* < 0.05). Patient cooperation, assessed using the Frankl scale, also improved significantly during the intervention period. Accommodation type demonstrated a significant association with OHI-S values, while diagnosis did not significantly influence the measured outcomes. Long-term follow-up demonstrated sustained improvements in oral hygiene and cooperation scores at both week 52 and week 104, indicating the potential long-term effectiveness of the preventive education program in children with special needs. **Conclusions:** The specific prevention program significantly improved oral health outcomes and cooperation among children with special needs.

## 1. Introduction

Accessibility is becoming increasingly important in today’s environment, especially for those with special needs. Children with special health care needs (SHCN) represent a highly heterogeneous population requiring individualized and multidisciplinary healthcare approaches. According to the American Academy of Pediatric Dentistry (AAPD), SHCN includes any physical, developmental, mental, sensory, behavioural, cognitive, or emotional condition requiring specialized medical management, healthcare interventions, or supportive services beyond routine care. These conditions may be congenital, developmental, or acquired, and often result in limitations affecting daily self-care activities and overall quality of life. Children with SHCN may include patients with developmental disorders such as cerebral palsy, cognitive impairments, autism spectrum disorder, congenital diseases, behavioural disorders, or systemic medical conditions. Due to their complex medical and functional needs, these individuals frequently require adapted dental care strategies and are considered at increased risk for oral diseases throughout their lifetime [1].

Given that 16% of the world population—roughly 1.3 billion people—experience significant disabilities, health disparities within this group must be addressed. These individuals face several challenges, such as poverty, stigma, discrimination, and barriers within the healthcare system. As a result, they are more likely to experience depression, asthma attacks, diabetes, strokes, obesity, and poor oral hygiene. Due to their dependence on caregivers and their irregular dental attendance, children with disabilities have higher rates of dental caries and periodontal problems, which raises the stakes significantly [2].

According to Alamri et al., clinical recommendations currently used for dentistry among children with special healthcare needs (SHCNs) are insufficient and of low quality. The scientific community should adopt evidence-based procedures and encourage cooperation between stakeholders to enhance these standards. The advantages of this are obvious: improved, more efficient guidelines can result in improved care for children with SHCNs, ensuring that they receive the high-quality dental treatment they need [3].

General anaesthesia (GA) is frequently used when treating young people who have trouble cooperating. However, there are hazards associated with GA that can negatively impact the general health of a patient. Paediatric dentists are advised to reserve GA for situations where it is impractical to perform routine dental procedures. Furthermore, there is evidence that GA and tooth trauma may be associated, ranging from small enamel fractures to severe avulsions. This risk is especially present during laryngoscopy, endotracheal intubation, or incorrect use of mouth openers [4,5].

Due to the small number of clinics offering GA covered by insurance, access to this procedure is restricted in Hungary, whereby law only anaesthesiologists can administer GA. As a result, waiting lists for children in need of GA for dental treatment can last for more than a year, significantly compromising their quality of life and making emergency dental care virtually impossible.

Children with cerebral palsy (CP) face severe scholastic obstacles due to the intricacy of the neurological disease, especially in reading, writing, and mathematics. Studies show that learning disabilities affect more than 59.5% of children with cerebral palsy. A child with cerebral palsy has a specific neuropsychological profile associated with these challenges, including severe working memory deficits and lower performance IQ. This highlights the necessity of providing these children with customized educational evaluations and interventions that address their cognitive and neuropsychological needs [6].

A new observational study launched in 2021 focuses on the oral health and behaviour of Hungarian children with cerebral palsy (CP) who were in the Conductive Education Program. The results show that children with CP had a higher prevalence of dental caries, particularly those in levels II and III of the Gross Motor Function Classification System (GMFCS). This research provides a basis for targeted interventions and preventive measures by using rigorous statistical analysis to identify significant associations between age, toothbrushing performance, and dental outcomes [7].

The present study was conducted in collaboration with the András Pető Faculty of Semmelweis University, an internationally recognized institution specializing in conductive education and the complex development of children with neurological and developmental disorders. The Pető method, developed by András Pető, is based on a holistic and interdisciplinary approach that combines pedagogy, rehabilitation, movement therapy, cognitive development, and social integration within a unified educational framework. Rather than focusing solely on physical impairments, conductive education aims to promote independence, motivation, self-sufficiency, and the development of “orthofunction,” enabling children to actively participate in daily life and society. Conductive education emphasizes active learning, neuroplasticity, group-based motivation, and task-oriented practice, supported by specially trained conductors who guide children through structured daily routines and functional activities. The method has primarily been applied in children with cerebral palsy and other neuromotor disorders, with evidence suggesting beneficial effects on motor performance, self-efficacy, and functional independence. Importantly, the educational environment of the Pető Institute is not limited exclusively to children with cerebral palsy. Children with various developmental, behavioural, cognitive, and communication difficulties also participate in conductive educational programmes. The structured daily routine, community-based learning environment, rhythmic intention techniques, and emphasis on motivation and social participation may provide benefits not only for motor development, but also for behavioural regulation, cooperation, attention, communication, and psychosocial functioning. This complex pedagogical approach may therefore offer advantages for a broader population of children with special educational and developmental needs [8,9].

The purpose of this pilot study is to test the hypothesis that children with special needs who participate in an eight-week special prevention program will experience improvements in both oral hygiene and dental treatment cooperation. As the term “special health care needs” represents a highly heterogeneous population, in the present study it was interpreted as children with motor coordination and/or intellectual impairments who required increased attention, support, and adaptation during dental care compared to typically developing children.

## 2. Materials and Methods

The target group of the study consisted of 97 school-age pupils from Pető András Conductive Practical Primary School, and parents were encouraged to participate via an online introductory presentation. The study was approved by the Hungarian Medical Research Council (TUKEB- IV/3957- 3/2022/EKU). Of the 20 children who initially returned signed consent forms, 16 were enrolled after assessment of their eligibility and general health status. Inclusion criteria were intentionally broad in order to minimize exclusion and to reflect the heterogeneity of children with special needs in everyday clinical practice. Participants were required to be between 7 and 21 years of age, attend the participating school, and have written parental or guardian consent.

From a motor-function perspective, the use of assistive devices such as wheelchairs, walking aids, or upper- and lower-limb orthoses was not considered an exclusion criterion. Children were eligible if they were able to move at least one upper limb independently or with assistance (e.g., supported positioning), and were capable of placing a toothbrush into their mouth. Excluded children with GMFS 5. They had to be able to swallow and spit their saliva on their own.

From an intellectual and behavioral perspective, participants had to be capable of at least minimal communication and partial self-care with or without assistance. Verbal communication was not mandatory; yes/no responses or communication via communication boards were considered sufficient.

Four children were excluded after the initial assessment. One child required urgent spinal surgery during the study period and was absent from school for several weeks. Two children were excluded because their intellectual and motor coordination abilities differed substantially from those of the other participants, making meaningful participation in the educational sessions difficult; these children were unable to communicate verbally, were difficult to engage in communication, and had difficulty following simple instructions. One additional child was excluded because the scheduled intervention sessions were not compatible with their timetable.

An eight-week dental preventive education program was created specifically for these children, with low to medium intellectual disabilities. The eight-week curriculum included subjects such as oral anatomy, pathology, best ways to brush teeth, and how to use dental instruments in a clinical setting. Children also had the opportunity to practice good oral hygiene and participate in hands-on dental instrument exploration. Interactive lectures, hands-on exercises, and interactive games were conducted by two dentists, one conductor, and one dental assistant. The eight-week program was followed by another eight-week period of no-intervention and no-supervision, during which children could follow their new routine and maintain their oral habits as they pleased.

The educational program consisted of eight structured sessions specifically tailored to the needs and attention span of children with special health care needs. The educational sessions were conducted over eight consecutive weeks and were originally planned to last approximately 45 min; however, 60 min time slots were scheduled to provide sufficient flexibility and avoid rushed teaching situations. This decision was made following consultation with the conductors working with the children daily, who emphasized that participants frequently became distracted, asked additional questions, or required repeated redirection of attention during educational activities.

The sessions were conducted by a multidisciplinary team consisting of two dentists, one dental student, and one conductor familiar with the children and their individual communication and behavioural needs. The conductor primarily acted as an observer and provided support when necessary. The first session focused exclusively on building trust and establishing rapport with the participants. Both educators and children introduced themselves according to their communication abilities, and informal discussions were initiated regarding oral hygiene habits and previous dental experiences. Personal stories and experiences were also shared by the educators to facilitate emotional connection and reduce anxiety associated with dental care.

The second session focused on basic oral anatomy, common oral diseases, and potential treatment options. Educational material was presented using PowerPoint (Version 16.103) presentations and specially designed demonstrational games to support understanding and maintain attention.

From the third session onward, each session began with an interactive revision activity using Kahoot-based quizzes. These activities proved highly motivating for the children and promoted active participation through a cooperative, game-based learning environment. Based on previous experience, the quizzes were intentionally designed to avoid clear winners and losers; instead, participants worked together to collect as many points as possible collectively. Children with communication difficulties were assisted using mobile communication applications, allowing them to actively participate in answering questions.

Following the revision activities, additional theoretical topics were introduced, including toothbrushing techniques, the importance and process of various dental treatments, oral hygiene tools, and dental instruments commonly encountered in dental offices. To reduce anxiety and increase familiarity, videos demonstrating dental instruments and procedures were prepared in advance. The sounds of dental equipment were repeatedly played during the sessions, and several playful educational exercises were incorporated, including blindfolded identification of instruments based on touch and recognition of dental instruments based on sound.

Two practical sessions were also incorporated into the program. During the first practical session, participants learned toothbrushing techniques adapted to their individual abilities. Children were asked to bring their own toothbrushes, which were individually evaluated regarding their advantages and limitations. After the use of plaque-disclosing tablets, the educators and conductor demonstrated the fundamentals of proper toothbrushing techniques to each child individually, primarily based on the modified Bass technique and adapted according to each participant’s motor abilities and personal needs.

The second practical session took place in the dental office environment, where children were introduced to previously discussed dental instruments and equipment in their original clinical setting. Participants were allowed to repeatedly handle and explore the instruments without the stress associated with actual dental treatment. Some children voluntarily tested selected instruments, such as the air–water syringe and saliva ejector, on themselves. The primary aim of this session was to increase familiarity with the dental environment and reduce fear related to future dental treatment.

Overall, the intervention consisted of one introductory session, three theoretical educational sessions, two practical sessions, and two dedicated revision sessions (on the 4th and 8th occasions). The revision sessions were considered particularly important to reinforce acquired knowledge, encourage further questions, and help children better understand the connections between oral hygiene practices and oral health outcomes.

Oral hygiene assessments were performed at multiple timepoints throughout the study. Baseline examinations were conducted before the initiation of the educational intervention (week 1). Follow-up assessments were subsequently performed immediately after completion of the active 8-week prevention program (week 8) and after an additional 8-week non-intervention period (week 16). Furthermore, long-term follow-up examinations were carried out in participants who were still attending the institution 1 year (week 52) and 2 years (week 104) after baseline assessment, allowing evaluation of the long-term sustainability of the intervention outcomes.

Two calibrated and blinded dentists followed the methods set by the World Health Organization (WHO) for dental evaluations and surveys. The assessments included comprehensive dental examinations according to WHO guidelines [10]. Oral hygiene was evaluated using a variety of oral hygiene indices, including the number of teeth with decay or fillings (as DF-T) [11] and the Oral Hygiene Index-Simplified (OHI-S) [12]. As it is impossible to differentiate between primary tooth changes and tooth loss associated with caries, the component “m” for missing teeth in deciduous dentition was omitted from the analysis. For the OHI-S measurement, a calibrated dentist recorded the amount of dental plaque and calculus covering the surface of the previously specified teeth. The Frankl Scale was used to record the level of cooperation during the examination [13].

Oral health examinations were performed in a dental office with artificial lighting and simple tools such as a dental probe and plain mouth mirror.

We determined the mean differences (MDs) with standard deviations (SDs) or 95% Confidence Interval (CI). For the sample size calculation, the analysis focused on the OHI-S index as the primary outcome measure. A mean change of 0.5 units from baseline was assumed as the expected intervention effect. Since no prior data were available, the standard deviation of the paired differences was estimated from the existing pilot data. Based on these parameters, a minimum of 27 participants would be required to detect the assumed effect size with adequate statistical power.

In addition, demographic parameters such as age, gender, and permanent residence were collected using a standardized questionnaire based on WHO guidelines. This table summarizes the demographic and clinical data of the 16 children included in the study. The table presents participant codes, age, primary diagnosis, Gross Motor Function Classification System (GMFCS) level, and accommodation type (home or dormitory). Most participants were diagnosed with cerebral palsy, while a smaller proportion presented primarily with behavioural problems without cerebral palsy. GMFCS levels were used to characterize the severity of motor impairment among participants with cerebral palsy. (Table 1) [10].

Conductive teachers completed dental care habit and oral health status questionnaires due to the school contexts of the children. These included frequency of tooth brushing, autonomy of toothbrushing, use of other brushing products (e.g., interdental brushes, etc.), oral habits, recent dental appointments including their reason and dietary habits. Involving the children into the data collection process provided us with invaluable insights into their everyday routines and school behaviours, adding to the comprehensiveness of our research.

The primary aim of the present pilot study was to evaluate the feasibility and practical applicability of the preventive educational program within the special educational environment. An additional objective was to identify potential difficulties and limitations arising during implementation in order to further optimize and refine the program before its possible integration into the regular school curriculum in the future.

In future studies, once the preventive education program becomes integrated into the school curriculum, we plan to apply a cyclical controlled study design. In this model, newly recruited groups would initially serve as control groups while participating in the same scheduled assessment sessions. Subsequently, these groups would receive the educational intervention and become intervention groups, while additional newly recruited participants would serve as controls.

This rotating approach would help preserve the ethical integrity of the study, as all participating children would ultimately benefit from the preventive program. At the same time, it would provide a more representative control population consisting of children with special needs from the same educational environment. Using healthy children as controls for a prevention program specifically designed for children with special needs would not be methodologically or clinically appropriate.

**Figure 1:**
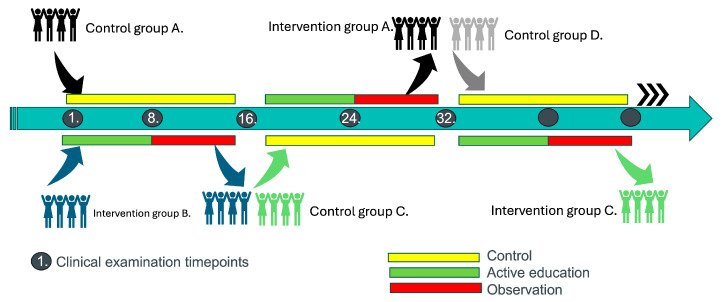
Proposed cyclical study design for future controlled implementation of the preventive education program.

Yellow bars indicate control periods, green bars represent active educational intervention phases, and red bars indicate observation periods without active education. Grey circles represent clinical examination timepoints (Figure 1).

Statistical analyses were performed using R statistical software (Version R 4.5.0) [14]. Descriptive statistics were calculated for all outcome variables and are presented as means with standard deviations (SDs) or mean differences (MDs) with 95% confidence intervals (95% CI), as appropriate.

To account for repeated measurements and within-subject correlation, linear mixed-effects models were applied, subject ID was included as a random intercept, while time, accommodation type, and diagnosis were included as fixed effects where applicable. Interaction terms between time and accommodation were explored in selected models.

All statistical tests were two-sided, and a *p*-value < 0.05 was considered statistically significant.

ChatGPT (OpenAI, San Francisco, CA, USA, GPT-5.5, accessed in 2026) was used for language editing, grammar checking, and improving the clarity of the manuscript. All AI-generated suggestions were carefully reviewed and verified by the authors.

## 3. Results

All participants attended more than 80% of the scheduled sessions. The mean DF-T index at baseline was 4.87 ± 3.36, and remained so throughout the active trial, as no dental care was provided during this 16-week-period.

A total of 16 children with special needs (mean age of 13.21 ± 2.04, 7 girls and 9 boys) participated in the preventive education program and completed the scheduled follow-up assessments. Changes in oral hygiene indices and cooperation scores were evaluated at baseline, week 8, week 16, week 52, and week 104 using linear mixed-effects models.

OHI-S values demonstrated significant improvement following the intervention. Compared to baseline, OHI-S scores were significantly lower at week 8 (Estimate = −1.4729, SE = 0.2917, t = −5.049, *p* = 9.89 × 10^−6^) and week 16 (Estimate = −1.4688, SE = 0.2573, t = −5.709, *p* = 1.58 × 10^−6^).

Similarly, DI-S scores showed significant reductions during the intervention period. DI-S values were significantly lower at week 8 (Estimate = −0.9088, SE = 0.1773, t = −5.126, *p* = 1.28 × 10^−5^) and week 16 (Estimate = −0.9292, SE = 0.1605, t = −5.789, *p* = 2.06 × 10^−6^) compared to baseline measurements.

CI-S values also improved significantly during the educational program. Compared to baseline, CI-S scores decreased significantly at week 8 (Estimate = −0.6129, SE = 0.1795, t = −3.414, *p* = 0.001308) and week 16 (Estimate = −0.5962, SE = 0.1588, t = −3.753, *p* = 0.000471).

Patient cooperation during dental examinations and oral hygiene procedures, assessed using the Frankl scale, also improved significantly during the intervention period. Frankl scores were significantly higher at week 8 (Estimate = 0.48817, SE = 0.19556, t = 2.496, *p* = 0.016464) and week 16 (Estimate = 0.46794, SE = 0.18010, t = 2.598, *p* = 0.012783) compared to baseline values.

At the 52-week follow-up, several improvements remained detectable compared to baseline. OHI-S values remained significantly lower at week 52 (Estimate = −0.7764, SE = 0.3035, t = −2.558, *p* = 0.01411), while CI-S values also remained significantly improved (Estimate = −0.4335, SE = 0.1863, t = −2.327, *p* = 0.024223). Frankl cooperation scores continued to show significant improvement at week 52 (Estimate = 0.18732, SE = 0.22644, t = 0.827, *p* = 0.412664). However, DI-S values no longer differed significantly from baseline at this timepoint (Estimate = −0.1709, SE = 0.2097, t = −0.815, *p* = 0.421).

At the 104-week follow-up, long-term improvements compared to baseline remained significant across multiple parameters. OHI-S values remained significantly lower than baseline (Estimate = −1.7315, SE = 0.2830, t = −6.119, *p* = 2.41 × 10^−7^). DI-S scores also remained significantly reduced at week 104 (Estimate = −1.1779, SE = 0.1857, t = −6.342, *p* = 3.29 × 10^−7^), while CI-S values continued to demonstrate significant improvement (Estimate = −0.6961, SE = 0.1737, t = −4.008, *p* = 0.000213). In addition, Frankl cooperation scores remained significantly higher compared to baseline values at week 104 (Estimate = 0.59401, SE = 0.20326, t = 2.922, *p* = 0.005518).

Table 2 presents the mean values and standard deviations (SDs) of oral hygiene indices measured at baseline (week 1), week 8, week 16, week 52, and week 104 follow-up examinations. The evaluated parameters included the Simplified Oral Hygiene Index (OHI-S), Debris Index-Simplified (DI-S), Calculus Index-Simplified (CI-S), and Frankl behaviour rating scale scores. The table also demonstrates the distribution of Frankl behaviour categories across the different examination timepoints, reflecting changes in patient cooperation during dental examinations and oral hygiene procedures throughout the study period.

**Figure 2:**
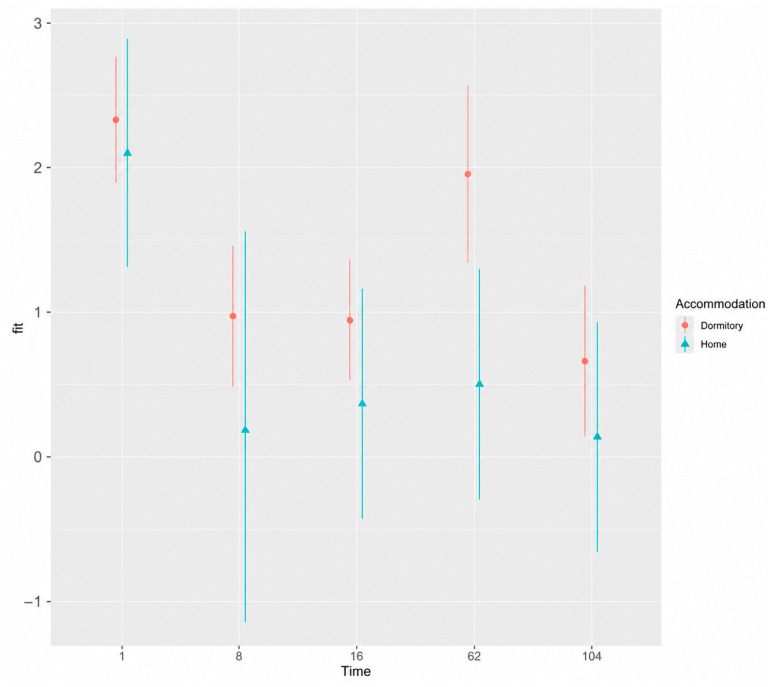
Longitudinal changes in OHI-S scores according to accommodation type.

The potential influence of diagnosis and accommodation type on treatment outcomes was also evaluated. Diagnosis did not demonstrate a significant effect on scores during follow-up assessments (*p* > 0.05). Accommodation type showed a significant association with OHI-S values, with children living at home demonstrating lower OHI-S scores compared to those living in institutional accommodation (Estimate = −0.6719, SE = 0.2276, t = −2.953, *p* = 0.00906). In the Frankl model, a significant interaction effect between accommodation type and week 52 measurements was observed (Estimate = −0.87041, SE = 0.40730, t = −2.137, *p* = 0.038327). (Figure 2).

## 4. Discussion

The present pilot study demonstrated that a tailored preventive oral health education program may improve oral hygiene and cooperation during dental treatment in children with special needs. Significant reductions were observed in OHI-S, DI-S, and CI-S scores following the intervention, while Frankl cooperation scores also improved during the active educational period. These findings suggest that structured, repeated, and individually adapted preventive education may positively influence both oral hygiene behaviour and patient cooperation in this vulnerable population. However, due to the pilot nature of the study, the relatively small sample size, and the absence of a control group, the findings should be interpreted cautiously.

The findings of the present study are consistent with previous investigations evaluating oral health promotion programs among children with cerebral palsy and other special health care needs populations. Maiya et al. demonstrated that preventive interventions combining powered toothbrushes and chlorhexidine spray significantly improved oral hygiene and gingival health parameters in children with cerebral palsy over a 6-week period [15]. Similarly, Vedha et al. reported that audiovisual parental oral health education resulted in significant improvements in OHI-S, plaque index, and gingival index scores compared to traditional educational methods. Our findings similarly support the concept that repeated and multimodal preventive education may contribute to measurable improvements in oral hygiene status in children with special needs [16].

An additional important finding of the present study was the improvement in cooperation during dental examinations and oral hygiene procedures. Children with special health care needs frequently experience difficulties during dental treatment because of communication barriers, anxiety, sensory hypersensitivity, impaired motor coordination, or cognitive limitations. Increasing familiarity with oral hygiene procedures and the dental environment may therefore contribute not only to improved oral health status but also to reduced stress and more successful dental experiences for both patients and clinicians. Similar findings have been described in studies emphasizing behavioural adaptation, repeated reinforcement, and caregiver-supported preventive education in special care dentistry [17,18].

The interactive and playful structure of the present educational program may also have contributed to the observed improvements in cooperation and oral hygiene behaviour. The use of repeated revision sessions, audiovisual educational materials, sound familiarization, and practical demonstrations allowed children to gradually become accustomed to dental instruments and procedures in a low-stress environment. Recent studies investigating app-based oral motor therapy and digital oral health interventions among children with cerebral palsy and autism spectrum disorders similarly reported that interactive educational approaches may improve oral hygiene practices, behavioural compliance, and engagement during oral care activities. The role of digital oral health interventions should also be considered in future preventive strategies for children with special health care needs. Recent evidence suggests that tailored mobile dental applications may improve oral hygiene practices, oral health awareness, cooperation during dental procedures, and caregiver involvement. Personalized m-health tools may be especially valuable for children with cognitive or motor impairments because they can adapt educational content, behavioural reinforcement, and familiarization techniques to the child’s individual sensory and communication needs. These technologies may therefore represent a promising adjunct to traditional preventive programs and could help reduce inequalities in access to oral health education and preventive care. Patient monitoring and teledentistry may represent promising future solutions for supporting oral hygiene maintenance in children with special health care needs, personal assistance and direct hands-on guidance during the initial stages of oral hygiene education remain essential and cannot be fully replaced [18,19].

The role of oral health literacy and caregiver involvement should also be emphasized when interpreting the findings of the present study. Children with cerebral palsy and other disabilities are frequently dependent on caregivers for daily oral hygiene procedures because of impaired dexterity and motor limitations. Previous studies demonstrated that limited oral health literacy among caregivers may negatively influence oral hygiene practices, oral healthcare-seeking behaviour, and long-term preventive outcomes in children with special health care needs. Furthermore, qualitative investigations have shown that caregivers often face substantial physical, emotional, and behavioural challenges while providing routine oral hygiene care for children with cerebral palsy. These observations may partially explain why sustained reinforcement and caregiver education appear essential for maintaining long-term improvements in oral health behaviour [17,20,21].

The present study also demonstrated differences associated with accommodation type. Children living at home showed more favourable OHI-S values than those living in institutional accommodation. These differences may partially reflect socioeconomic inequalities, differences in caregiver involvement, variability in oral health literacy, or differences in access to supervised daily oral hygiene support. Many institutionalized children originate from socioeconomically disadvantaged backgrounds, where preventive dental care and oral health education may be more limited. Previous public health studies have similarly emphasized the importance of community-based preventive models and caregiver-supported oral health promotion programs for reducing oral health disparities among children with special health care needs [22].

An important aspect of the present intervention was its school-based and community-oriented structure. The educational sessions were integrated into the children’s familiar educational environment and involved close collaboration with conductors and school personnel. Similar school-based oral health promotion programs among children with cerebral palsy demonstrated that participatory and environmentally integrated approaches may improve oral hygiene outcomes over longer follow-up periods. Songsiripradubboon and Krisdapong reported significant long-term improvements in plaque scores during an 18-month school-based oral health promotion program that combined education, staff training, policy integration, and community participation. These findings support the concept that oral health promotion in children with disabilities may benefit from broader environmental and institutional support rather than isolated educational interventions alone [23].

Although significant improvements were observed during the active intervention phase, some parameters demonstrated partial deterioration following the non-intervention period. This finding may indicate that oral hygiene habits acquired during preventive programs require continuous reinforcement to remain sustainable over time. Nevertheless, several improvements remained detectable during the 52-week and 104-week follow-up examinations, suggesting that the intervention may still provide long-term beneficial effects despite some degree of behavioural relapse. Similar observations have been reported in previous oral health promotion studies, where repeated reinforcement and regular follow-up sessions were considered essential for maintaining long-term behavioural changes in vulnerable pediatric populations [23].

### 4.1. Strengths

By focusing on the efficacy of a customized dental prevention program for young patients with special needs, explicitly addressing oral health and cooperation during dental procedures, the study effectively fills in a gap in the literature. Improving oral hygiene can be approached through the comprehensive design of the program, which combined educational and practical components. An additional guarantee that the study findings were solid and consistent with previous research was the use of validated WHO protocols and indices. Further confirming the potential for wider use of the program, the intervention showed notable improvements in important oral health indices.

Several measures were implemented to reduce potential sources of bias. To minimize selection bias, inclusion and exclusion criteria were intentionally kept broad in order to reflect the heterogeneous population of children with special needs encountered in everyday clinical practice. All participants received the same educational intervention according to a standardized protocol delivered by the same trained professionals. Clinical examinations and oral hygiene assessments were performed using standardized indices and calibrated evaluation methods to reduce measurement bias. Attrition bias was minimized by conducting the program within the school environment and coordinating the intervention sessions with teachers and caregivers whenever possible. Nevertheless, due to the pilot nature of the study, the relatively small sample size and the absence of a control group may still have introduced potential bias and limited the generalizability of the findings.

### 4.2. Limitations

As this was a pilot study involving a small number of participants, the findings should be interpreted with caution. Nevertheless, the positive trends observed indicate that the intervention is feasible and potentially effective. We acknowledge that the relatively small sample size is a limitation of the present pilot study and may reduce the statistical power and generalizability of the findings. Although the calculated minimum sample size was 27 participants, this number could not be achieved due to the school’s organizational limitations and fixed educational schedule. Nevertheless, the primary aim of this pilot study was to evaluate the feasibility and preliminary effectiveness of the prevention program in a real-world special education setting. In the future, our goal is to integrate the preventive education program into the regular school curriculum, enabling children to participate in the full educational program from the lower grades onward. As students progress to higher grade levels, they would receive reinforcement and revision sessions rather than the complete introductory program again. Through this stepwise approach, all children attending the institution could gradually benefit from the intervention.

Several potential confounding factors may have influenced the outcomes of the present study. These included differences in the participants’ intellectual abilities, motor coordination, baseline oral hygiene status, level of caregiver support, communication skills, and degree of cooperation during dental procedures. Variability in home oral hygiene practices, previous dental experiences, concomitant dental treatments, and general medical conditions may also have affected the observed results. Due to the heterogeneity of children with special needs, complete control of these factors was not possible within the framework of this pilot study.

Another limitation of the present study is that the participant population primarily consisted of children with cerebral palsy; therefore, the findings may not be fully generalizable to the broader population of children with other types of special health care needs.

### 4.3. Implications for Practice

This study highlights the significance of developing customized oral health programs for children with disabilities, such as cerebral palsy. Dentists should prioritize early, planned training initiatives that focus on improving oral hygiene practices and encouraging cooperation during dental surgery. In terms of the significant improvements in oral health indicators, implementing similar initiatives in clinical and educational contexts can improve the general dental care experience for children with specific health care needs. The study also highlights how behaviours tend to revert over time without reinforcement, so ongoing support is critical to maintain the gains made through these strategies.

### 4.4. Implications for Research

Future research should focus on expanding the scope of this study, with larger sample sizes and control groups, to validate the findings in a wider population of children with special needs. In addition, longer follow-up periods are necessary to assess the sustainability of oral health and cooperation improvements. Research should also explore the integration of multimedia tools, such as video-based education, to see if these can further increase engagement and long-term habit retention. Investigating the effectiveness of combining educational interventions with mechanical or chemical treatments (e.g., powered toothbrushes or chlorhexidine) will be crucial in developing more comprehensive, long-term dental care strategies for children with disabilities.

## 5. Conclusions

Within the limitations of this pilot study, the findings suggest that a tailored preventive oral health education program may contribute to improved oral hygiene and cooperation during dental treatment in children with special needs. Significant short-term improvements were observed in several oral hygiene indices and behavioural cooperation scores, while some positive effects remained detectable during long-term follow-up assessments.

However, due to the relatively small sample size and the absence of a control group, the results should be interpreted cautiously and cannot be generalized to the wider population of children with special needs. The findings primarily demonstrate the feasibility and potential effectiveness of implementing structured preventive oral health programs within special education settings, but further controlled studies with larger sample sizes are necessary to confirm the preliminary results.

## Figures and Tables

**Table 1:** Demographic and clinical characteristics of the study participants.

Code	Age (Years)	Diagnosis	GMFS	Accommodation
4SZK	13	Cerebral palsy	4	Dormitory
2DE	16	Cerebral palsy	2	Dormitory
4SZC	14	Cerebral palsy	2	Dormitory
4LA	13	Cerebral palsy	3	Home
2SZS	17	Cerebral palsy	2	Dormitory
2NK	16	Cerebral palsy	2	Dormitory
4VÁ	14	Cerebral palsy	4	Home
4SZR	12	Behaviour problems	No cerebral palsy	Dormitory
3NO	11	Behaviour problems	No cerebral palsy	Home
3GYT	12	Cerebral palsy	2	Home
2SR	16	Cerebral palsy	1	Dormitory
3SZP	11	Cerebral palsy	3	Dormitory
2GO	16	Behaviour problems	No cerebral palsy	Home
3NE	14	Cerebral palsy	4	Dormitory
3TCS	11	Cerebral palsy	3	Dormitory
2CSSZ	14	Cerebral palsy	4	Dormitory

**Table 2:** Longitudinal changes in oral hygiene indices and cooperation scores during the follow up period.

	1-Week (Baseline)		8-Week		16-Week		52-Week		104-Week	
n	15		10		16		8		10	
	Mean	SD	Mean	SD	Mean	SD	Mean	SD	Mean	SD
OHI-S	2.35	0.74	0.85	0.61	0.88	0.61	1.40	1.06	0.49	0.57
DI-S	1.41	0.44	0.55	0.55	0.54	0.36	0.96	0.68	0.28	0.37
CI-S	0.94	0.63	0.30	0.23	0.34	0.29	0.44	0.43	0.19	0.24
Frankle										
2	2	13%	0	0%	0	0%	0	0%	0	0%
3	8	53%	1	10%	3	19%	0	0%	0	0%
4	5	33%	9	90%	13	81%	8	100%	10	100%

## Data Availability

The data that support the findings of this study are available upon request from the corresponding author. The data are not publicly available due to privacy or ethical restrictions.

## Abbreviations

The following abbreviations are used in this manuscript:

CP	Cerebral Palsy
SHCN	Special healthcare needs
GA	General anaesthesia
GMFCS	Gross Motor Function Classification System
DF-T	Decayed/Filled-Teeth
OHI-S	Oral Hygiene Index—Simplified
MD	Mean Difference
SD	Standard Deviation
CI	Confidence Interval
